# Fluid Management in Veno-Arterial Extracorporeal Membrane Oxygenation Therapy—Analysis of an Experimental Pig Model

**DOI:** 10.3390/jcm12165330

**Published:** 2023-08-16

**Authors:** Ilija Djordjevic, Johanna Maier-Trauth, Stephen Gerfer, Mara Elskamp, Thomas Muehlbauer, Alexandra Maul, Pia Rademann, Borko Ivanov, Ihor Krasivskyi, Anton Sabashnikov, Elmar Kuhn, Ingo Slottosch, Thorsten Wahlers, Oliver Liakopoulos, Antje Christin Deppe

**Affiliations:** 1 Department of Cardiothoracic Surgery, University Hospital Cologne, 50937 Cologne, Germany; 2 Division of Thoracic and Cardiovascular Surgery, HELIOS Klinikum Siegburg, 53721 Siegburg, Germany; 3 Faculty of Medicine, University Hospital of Cologne, Experimental Medicine, University of Cologne, 50937 Cologne, Germany; 4 Department of Cardiothoracic Surgery, Otto-von-Guericke University Magdeburg, 39106 Magdeburg, Germany; 5 Department of Cardiac Surgery, Kerckhoff-Clinic Bad Nauheim, Campus Kerckhoff, University of Giessen, 35392 Giessen, Germany

**Keywords:** ECMO, fluid therapy, fluid overload

## Abstract

(1) Background: Fluid resuscitation is a necessary part of therapeutic measures to maintain sufficient hemodynamics in extracorporeal membrane oxygenation (ECMO) circulation. In a post-hoc analysis, we aimed to investigate the impact of increased volume therapy in veno-arterial ECMO circulation on renal function and organ edema in a large animal model. (2) Methods: ECMO therapy was performed in 12 female pigs (Deutsche Landrasse × Pietrain) for 10 h with subsequent euthanasia. Applicable volume, in regard to the necessary maintenance of hemodynamics, was divided into moderate and extensive volume therapy (MVT/EVT) due to the double quantity of calculated physiologic urine output for the planned study period. Respiratory and hemodynamic data were measured continuously. Additionally, renal function and organ edema were assessed by blood and tissue samples. (3) Results: Four pigs received MVT, and eight pigs received EVT. After 10 h of ECMO circulation, no major differences were seen between the groups in regard to hemodynamic and respiratory data. The relative change in creatinine after 10 h of ECMO support was significantly higher in EVT (1.3 ± 0.3 MVT vs. 1.8 ± 0.5 EVT; *p* = 0.033). No major differences were evident for lung, heart, liver, and kidney samples in regard to organ edema in comparison of EVT and MVT. Bowel tissue showed a higher percentage of edema in EVT compared to MVT (77 ± 2% MVT vs. 80 ± 3% EVT; *p* = 0.049). (4) Conclusions: The presented data suggest potential deterioration of renal function and intestinal mucosa function by an increase in tissue edema due to volume overload in ECMO therapy.

## 1. Introduction

Extracorporeal membrane oxygenation (ECMO) is frequently used in clinical practice for various indications [1]. The initial phase of ECMO therapy might be difficult to manage because of inadequate ECMO flow and severe hemodynamic decline [2,3]. Fluid resuscitation is a necessary part of therapeutic measures to maintain sufficient hemodynamic and ECMO circulation. Pathophysiologic conditions, such as shock, low cardiac output, and sepsis-like syndromes, additionally influence the requirement for volume administration [4]. Moreover, increased capillary leakage is known to contribute to a higher demand for fluids [5]. Several sepsis studies demonstrated the negative impact of excessive volume administration on the outcome of these patients [6,7,8,9,10,11]. In contrast, only a few clinical reports shed some light on the specifics of ECMO therapy and the impact of fluid resuscitation on patient outcomes [12,13,14]. In this regard, fluid overload was identified as having a negative impact on outcomes, especially in pediatric ECMO patients [15]. For adult veno-venous ECMO patients, a negative cumulative daily fluid balance was associated with improvement of respiratory function [16]. Therefore, balanced fluid management in ECMO patients is crucial to avoiding adverse outcomes. In this regard, several strategies are in use to assess fluid requirements in critically ill adults, such as fluid challenge [17], inferior vena cava assessment [18,19], passive leg raising [20], and end-expiratory occlusion maneuver [21]. 

A particular focus of the underlying study was to investigate the impact of increased volume therapy in ECMO circulation on organ edema by analyzing retrospective data from a large animal experimental protocol performed by our study group. 

## 2. Materials and Methods

The study performed is a retrospective, not pre-planned, subanalysis of one particular study arm, which was part of a large animal experimental protocol investigating concomitant extracorporeal membrane oxygenation (ECMO) and intra-aortic balloon pumping (IABP) and was approved by the responsible ethical committee for animal care of the University of Cologne (LANUV, Bonn, Northrhine-Westphalia, Germany) [22]. Data were prospectively collected. The Koeln Fortune Program (343/2015), Faculty of Medicine, University of Cologne, funded this research. The experimental protocol and subsequent analysis were performed between May 2017 and December 2021 at the Experimental Medicine institute of the Faculty of Medicine Cologne and the Department of cardiothoracic surgery of the University Hospital Cologne. The study arm investigated in this report included twelve healthy pigs with ECMO circulation for 10 h.

### 2.1. Experimental Protocol

Twelve female pigs (Deutsche Landrasse × Pietrain) were included in this study. Animals were premedicated with xylazin (2 mg/kg), tiletamin (10–12.5 mg/kg i.m.), and zolazepam (10–12.5 mg/kg i.m.). Deep sedation was induced with the intravenous application of propofol (1.5–2.5 mg/kg). Neuromuscular blockade was achieved with pancuronium (0.2 mg/kg i.v.). After endotracheal intubation, continuous anesthesia and analgesia were maintained with the intravenously administered propofol (4–6 mg/kg/h), midazolam (0.96–1.2 mg/kg/h), and fentanyl (0.012–0.025 mg/kg/h). Animals were ventilated with volume-controlled ventilation (Fabius GS, Dräger, Luebeck, Germany) using an air/oxygen mixture adjusted to keep arterial blood gas values in the physiologic range (partO_2_ 100–150 mmHg, partCO_2_ 35–45 mmHg, pH 7.35–7.45). 

### 2.2. Placement of Measurement Catheters

Appropriate parts of the body were sterilized. A urine catheter was placed surgically into the bladder. The body temperature was measured through a rectal catheter. Afterwards, the right jugular vein and carotid artery were cannulated for measurements of the central venous pressure (8.5 Fr., Arrow International, Reading, PA, USA), the mean arterial pressure (20 G Leadercath, Vygon, Aachen, Germany), and for blood sampling. A contralateral carotid artery was prepared, and carotidal blood flow was measured by a transit time flow probe (Transonic Systems, Inc., New York, NY, USA). After sternotomy and pericardial incision, the pulmonary artery was cannulated for blood sampling and for pulmonary artery measurements (20 G Leadercath, Vygon, Aachen, Deutschland). Pressure transducertip catheters (Model SPC-350S, Millar Instruments, Inc., Houston, TX, USA) were placed into the right and left ventricles. Cardiac output (CO) was recorded by a transit time flow probe (Transonic Systems, Inc., New York, NY, USA) placed around the ascending aorta. 

**Figure 1:**
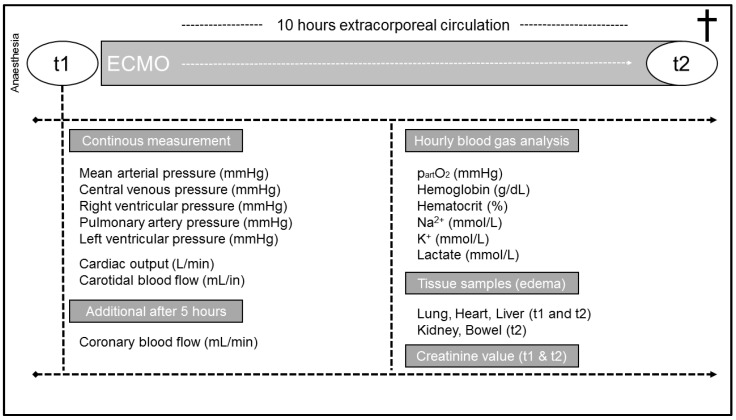
Flow chart of the study protocol and assessed parameters.

ECMO cannulas (HLS cannula, Getinge, Rastatt, Germany) were implanted through groin vessels (arterial: 17 Fr.; venous: 21 Fr.). Heparin was applied intravenously as an anticoagulant drug. Extracorporeal circulation (BIO MEDICUS Medtronic 540 Bio Console, Medtronic, Dublin, Ireland) was started after initial measurements and sampling of baseline parameters. An additional heater (BIO MEDICUS Medtronic BIO-CAL 370, Medtronic, Dublin, Ireland) was implemented into the ECMO circuit. The study protocol ended 10 h after the initiation of ECMO therapy (Figure 1). Animals were euthanized by applying an overdose of pentobarbital (80 mg/kg). 

### 2.3. Hemodynamic Measurements

Hemodynamic measurements were performed during stable hemodynamic and respiratory conditions before extracorporeal circulation (t1) and 10 h after initiation of ECMO circulation (t2). All data were digitalized at a rate of 500 Hz using a 16-channel hemodynamic set-up and subsequently analyzed (Hugo Sachs Elektronik-Harvard Apparatus GmbH, March-Hugstetten, Germany). 

### 2.4. Laboratory Parameters

Blood gas analysis was performed hourly. Additionally, creatinine values were sampled and analyzed for t1 and t2.

### 2.5. Target Parameters and Settings during ECMO Circulation

Particular hemodynamic and respiratory parameters during ECMO circulation were targeted as follows:

ECMO-flow → 50 mL/kg/min/m^2^ (indexed)

MAP → 60–70 mmHg

p_art_O_2_ → 120–200 mmHg

### 2.6. Fluid Resuscitation during ECMO Circulation

**Figure 2:**
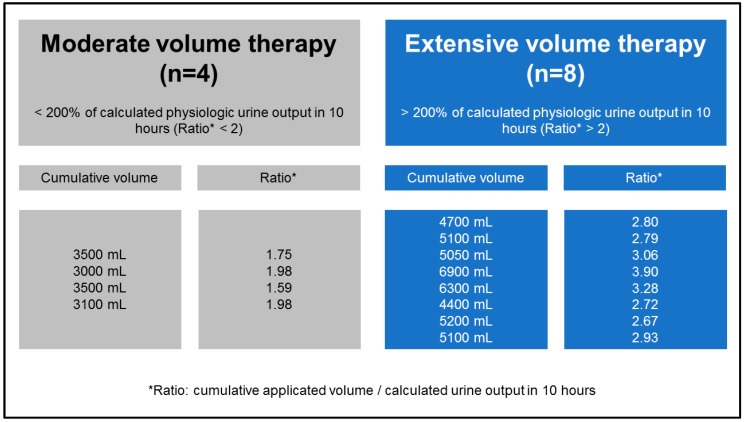
Presentation of groups after dichotomization in moderate and extensive volume therapy.

To maintain targeted mean arterial pressure, a fluid resuscitation protocol was tracked with a ratio of colloids and crystalloids of approximately 1:3. The study protocol strictly excluded blood transfusions. As the animals included in the previous investigation were retrospectively analyzed, the full population was divided into pigs who received moderate volume therapy and those receiving extensive volume therapy (Figure 2). The criteria used for allocating pigs to cohorts was the scaling of volume application, which was based on the double quantity of calculated physiologic urine output (0.05 mL/kg/min) for the planned study period [23].

### 2.7. Organ Edema

The primary outcome of this study was the relationship between organ edema. Tissue samples of the lung, heart, and liver were obtained at t1 and t2, and tissue samples of the kidneys and bowel were obtained only at t2. Probes were weighed (wet weight) and then freeze-dried after lyophilization. Dry weights were measured after the incubation of specimens at 110 °C for 2 days and corrected for residual water content. Organ edema was calculated by using the formula: % tissue water = (wet weight dry weight) wet weight and expressed as a percentage of the wet weight. All measurements were performed in duplicate, and the average was taken to balance the influence of hydrostatic distribution on the different organ tissue water contents.

### 2.8. Statistical Analysis

Statistical analysis was performed using Statistical Package for Social Sciences, version 23.0 (SPSS IBM, Chicago, IL, USA). Data were evaluated for normality using one sample Kolmogorov-Smirnov test and expressed as the mean ± standard deviation. Univariate analysis was performed using either the Student t or Mann-Whitney U test for normally and non-normally distributed continuous variables, respectively. *p*-values < 0.05 were considered statistically significant.

## 3. Results

**Figure 3:**
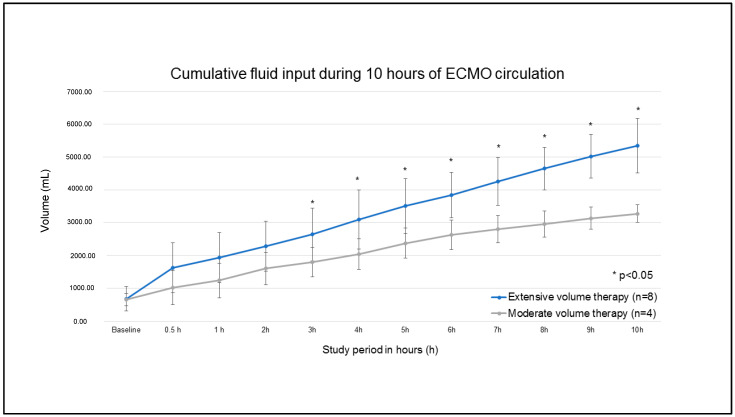
Cumulative fluid input during 10 h of ECMO circulation for the EVT and MVT groups.

After dichotomization of fluid administration within the study period, four pigs were included in the moderate volume therapy (MVT) group, and eight pigs were analyzed in the extensive volume therapy (EVT) group (Figure 2). Cumulative volume administration differed significantly after the third hour of ECMO circulation (Figure 3). Cumulative fluid administration after 10 h of ECMO circulation (t2) was significantly different (3275 ± 263 mL MVT vs. 5344 ± 834 mL EVT; *p* < 0.01). 

### 3.1. Hemodynamics, Respiratory Data, and Blood Gas Analysis 

**Figure 4:**
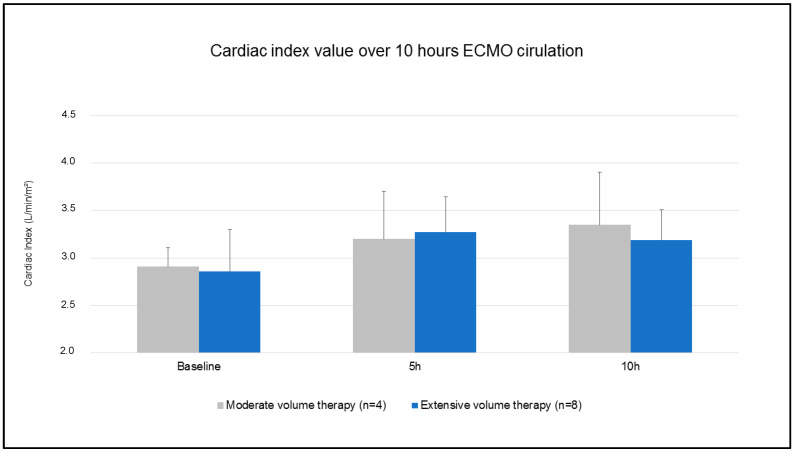
Cardiac index value over 10 h ECMO circulation.

Baseline data are summarized in Table 1. Hemodynamic parameters were comparable between the groups at t1 (cardiac output: 3.1 ± 0.2 L/min MVT vs. 3.0 ± 0.5 L/min EVT; *p* = 0.666/MAP: 71 ± 9 mmHg MVT vs. 66 ± 11 mmHg EVT; *p* = 0.417/CVP: 12 ± 1 mmHg MVT vs. 13 ± 4 mmHg EVT; *p* = 0.683), except for a higher mean pulmonary arterial pressure in MVT (25 ± 1 mmHg MVT vs. 23 ± 3 mmHg EVT; *p* = 0.041). Additionally, respiratory data showed no statistical difference. FIO_2_ did not differ between the groups. The cardiac index was adjusted in regard to the targeted values and showed no difference between the groups during the study period (Figure 4). After 10 h of ECMO circulation, cumulative urine output did not differ between the groups (776 ± 495 mL MVT vs. 1003 ± 504 mL EVT; *p* = 0.484). Hemodynamic data showed no difference in accordance with the volume applied after 10 h of ECMO therapy (Table 2). Blood flow in the ascending aorta decreased significantly in both groups, with no difference between MVT and EVT (0.2 ± 0.1 L/min MVT vs. 0.5 ± 0.4 L/min EVT; *p* = 0.128). Carotidal and coronary flow were comparable. Moreover, MVT did not impact respiratory data (Table 2). Blood gas analysis showed a significant higher decrease of hemoglobin (7.9 ± 0.4 g/dL MVT vs. 6.7 ± 1.2 g/dL EVT; *p* = 0.035) and haematocrit (25 ± 1% MVT vs. 21 ± 4% EVT; *p* = 0.036) in EVT. Lactic acid levels were higher in EVT.

### 3.2. Relative Creatinine Change

**Figure 5:**
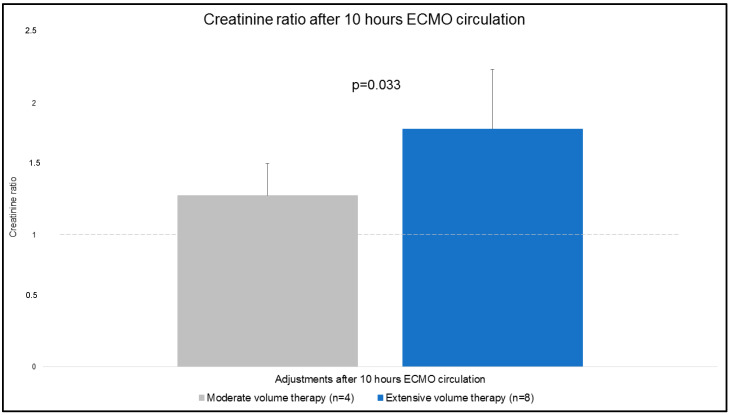
Creatinine ratio after 10 h ECMO circulation.

Creatinine values were obtained at t1 and t2. Relative changes (creatinine ratio) of absolute values for both groups are presented in Figure 5. The creatinine ratio was significantly higher in EVT compared to MVT (1.3 ± 0.3 MVT vs. 1.8 ± 0.5 EVT; *p* = 0.033).

### 3.3. Organ Edema

**Figure 6:**
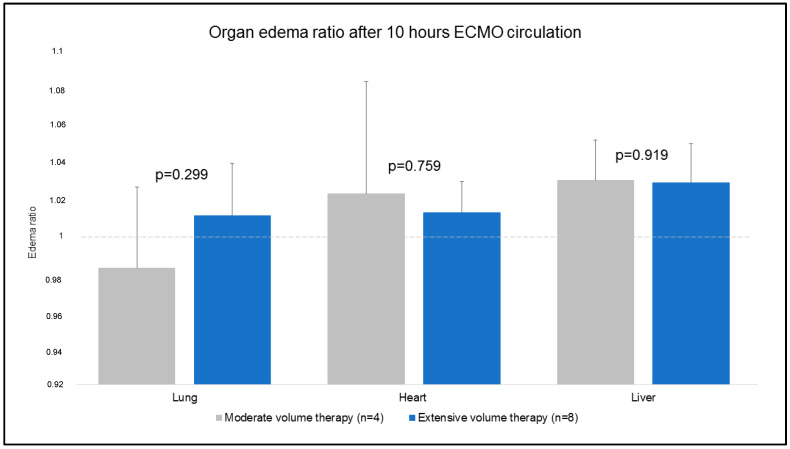
Organ edema ratio after 10 h ECMO circulation.

**Figure 7:**
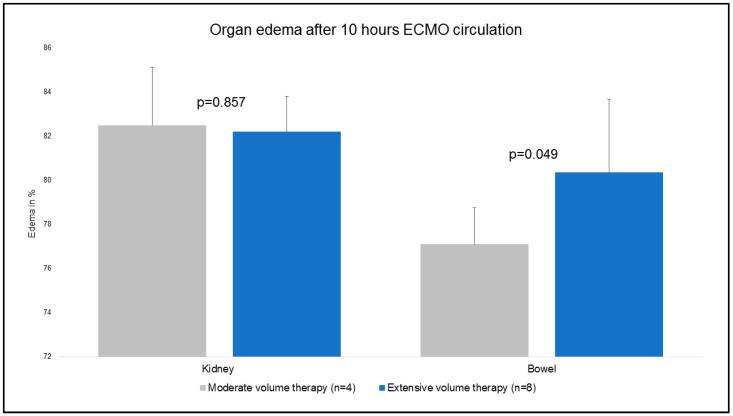
Organ edema (%) after 10 h ECMO circulation.

Tissue probes of the lung, heart, and liver were obtained at t1 and t2. The primary outcome showed that no major differences were evident for the mentioned organ samples in regard to the organ edema ratio (Figure 6). Due to the experimental protocol, tissue samples of the kidney and bowel were taken at t2 (Figure 7). Kidney samples were comparable (83 ± 3% MVT vs. 82 ± 2% EVT; *p* = 0.857). Bowel tissue showed a higher percentage of edema in EVT compared to MVT (77 ± 2% MVT vs. 80 ± 3 % EVT; *p* = 0.049).

## 4. Discussion

The main findings of our analysis investigating the impact of volume overload during ECMO circulation were:

- From our analysis excessive fluid resuscitation might affect intestinal tissue structure and contribute to dysfunction of the intestinal barrier.
- Higher renal dysfunction in EVT was shown.

### 4.1. Clinical Studies Indicating the Association of Fluid Overload with Adverse Outcomes

The reasons for fluid overload in ECMO patients are heterogeneous and multifactorial. In clinical practice, fluid administration in ECMO patients frequently depends on intravascular hypovolemia, which causes the suction phenomenon of the ECMO circuit. Moreover, maintenance of targeted mean arterial pressure (>60 mmHg) is achieved by simultaneous vasopressor and volume therapy. In patients with compromised hemodynamics, initial excessive volume application might stabilize the hemodynamic situation and result in suction-free ECMO circulation. On the other side, this overload might negatively impact the following days’ ECMO support. Schmidt and colleagues from Paris and Melbourne were the first to report the impact of fluid balance on outcome after ECMO therapy [12]. In their retrospective analysis of 172 patients treated with either veno-arterial or veno-venous ECMO, the authors could prove that early positive fluid balance at day 3 after ECMO initiation was an independent predictor of 90-day mortality, regardless of primary diagnosis, acute kidney injury (AKI), or required hemodialysis. 

Another study group by Staudacher investigated 195 patients, showing that higher fluid balance was significantly associated with less survival in ECMO patients and might be useful for prognostication [13]. In regard to the outcome, the authors found no evidence supporting liberal volume therapy. Kim and colleagues from South Korea investigated 723 ECMO patients retrospectively and reported on a significantly increased risk of 90-day mortality in patients with higher fluid balance values, corroborating the findings of Schmidt and Staudacher [14]. The authors presented that mortality risk did not increase until a fluid balance of 82.3 mL/kg was reached in patients with cardiovascular causes for ECMO. In patients with pulmonary indications for ECMO, levels above 189.6 mL/kg were associated with an increase in mortality [14]. Additionally, Anton-Martin et al. recently presented congruent results for neonatal and pediatric ECMO patients in their observational retrospective analysis [24]. Fluid accumulation within the first day after ECMO implementation was significantly associated with increased ICU mortality, showing a higher proportion of AKI in non-survivors. These findings are consistent with the data provided by Selewski and colleagues [25]. Selewski investigated 756 children under ECMO support and showed that fluid overload was independently associated with adverse outcomes, including increased mortality and increased duration of extracorporeal membrane oxygenation [25]. 

Interestingly, in our analysis, lactate levels differed between the groups. The fact that the hemoglobin differed might contribute to these circumstances. However, hemoglobin differences are expressed by fluid overload. It is speculative if low hemoglobin concentrations affected lactate levels or if the proven renal and intestinal dysfunction was followed by a lactic acid increase.

Moreover, a fluid overload might lead to distension of the left ventricle, and physicians should be aware of such a complication. Actually, there are some strategies for ventricular unloading (Impella, IABP), and the right one depends on several parameters.

### 4.2. Excessive Volume Administration Might Trigger AKI in ECMO Patients

As fluid administration is essential for outcomes in ECMO patients, Bridges and colleagues recently published guidelines for fluid and electrolyte management in ECMO patients, with special attention to the development and therapeutic options of AKI [26]. The authors clearly stated that fluid overload and AKI are associated with increased morbidity and mortality in ECMO patients. These clinical findings are supported by our analysis. In comparison of MVT and EVT, our data showed that creatinine levels were significantly higher in EVT, suggesting a deterioration of renal function, whereas cumulative urine output did not differ between the groups (776 ± 495 mL MVT vs. 1003 ± 504 mL EVT; *p* = 0.484). As our volume administration was based on targeted cardiac index and mean arterial pressure, fluid overload in EVT might affect end organ function. In translation to clinical settings, a targeted higher ECMO blood flow will result in more frequent ECMO suction events and thereby trigger volume therapy in non-hypovolemic patients. This iatrogenic-induced hypervolemia is known to affect renal function. Recently, Murphy and colleagues reported on a multicenter study analyzing the impact of AKI, fluid overload, and renal replacement therapy on mortality in neonatal ECMO patients [27]. The authors showed that fluid overload (OR 1.20, 95% CL 1.11–1.30) and acute kidney injury requiring hemodialysis (OR 2.12, 95% CL 1.20–3.73) were independently associated with mortality [27]. 

Moreover, ECMO circulation itself is known to induce a systemic inflammatory response, requiring fluid resuscitation. In addition, ECMO has been described as a risk factor for AKI [28]. In clinical scenarios, AKI was shown to occur in 35.5–74.0% of patients treated with ECMO due to cardiogenic shock and cardiac arrest [29]. The contribution to AKI by ECMO is explainable by exposure to foreign membranes, non-pulsatile renal blood flow, increased blood shear stress, air and blood embolisms, hemolysis, and organ crosstalk of the heart, lung, and kidneys [30]. Yuan et al. demonstrated in an experimental cardiac arrest model that ECMO might alleviate AKI by downregulating AKI-related gene expression and apoptosis after cardiac arrest [31]. Further investigation is necessary to evaluate and translate the described findings.

To avoid the negative impact of fluid overload in ECMO patients, Thomas et al. suggest initiating in an early phase continuous renal replacement therapy (RRT) targeting in this way a negative fluid balance [32]. The authors showed that the patient groups with ECMO support and RRT with a negative fluid balance (−3840 mL vs. +425 mL; *p* ≤ 0.005) were associated with higher survival to discharge rates. In general, indications to implement RRT are varying. A multicentric survey of ECMO centers showed that the most frequent indications for RRT in ECMO patients were fluid overload (43%), the prevention of EVT (16%), and the occurrence of AKI (35%) [33]. The technical implementation of RRT in on-going ECMO support, whether directly into the ECMO circulation or in parallel, is a matter of current discussion [34]. In accordance with the published guidelines for fluid and electrolyte management in ECMO patients, early initiation of RRT should always be an integral part of therapeutic considerations to achieve solute clearance, adjust electrolyte discrepancies, and prevent EVT [26]. 

### 4.3. Impact of Excessive Fluid Resuscitation on Intestinal Edema and Function

As already mentioned, fluid overload is known to impact organ function. In our analysis, organ edema was assessed and analyzed in comparison to MVT and EVT. Interestingly, organ edema in intestinal tissue samples showed significantly higher manifestation in EVT (77 ± 2% MVT vs. 80 ± 3% EVT; *p* = 0.049). Impact on several other tissue samples (lung, heart, liver, and kidneys) was not shown. 

In regard to fluid overload-induced intestinal microcirculatory damage, Behem and colleagues from Hamburg (Germany) reported on their large animal study investigating the impact of volume therapy on intestinal microcirculation [35]. They showed that ischemia-reperfusion led to impaired intestinal microcirculation, which was not restored by volume administration despite improved cardiac output [35]. In this regard, volume therapy should be titrated with meticulous care, particularly after ischemia-reperfusion injury, and ECMO therapy should be implemented in extracorporeal cardiopulmonary resuscitation. Additionally, unnecessary fluid loads may impair microcirculatory flow and worsen tissue edema, as we showed with our data. Several molecular-biological pathways are discussed to trigger an increase in edema in intestinal tissue; however, this particular point was not assessed in our study and needs further investigation. Kurundkar and colleagues presented results from their experimental study, showing an ECMO-induced rapid increase in gut mucosal permeability within the first 2 h of treatment, leading to a significant rise in circulating bacterial products [36]. Gut barrier dysfunction might be triggered in this way by ECMO, explaining the potential for plainer bacterial translocation. In contrast to the findings of Kurundkar, Liang and colleagues showed in their large-animal experimental protocol (CPR + ECMO vs. ECMO, n = 24) that intestinal mucosal epithelial cells in the CPR + ECMO group exhibited significantly reduced congestion and edema in comparison with those in the CPR group [37]. Moreover, expression of anti-apoptotic proteins in intestinal mucosa was higher in the ECMO + CPR group. In this regard, Ni et al. demonstrated in their pig-model (n = 21) that ECMO support aggravates intestinal mucosal injury early during ECMO support. However, protective effects on the intestinal mucosal barrier were shown 24 h after ECMO initiation, indicating a positive impact at a later stage of ECMO circulation [38]. He and colleagues provided an interesting study investigating the impact of RRT on the intestinal mucosal barrier during ECMO circulation [39]. 24 pigs were included in this study, and histopathologic findings showed that ECMO-induced intestinal edema and morphological distortion of epithelial cell structures were significantly improved by adding RRT to ECMO therapy. This finding supports the hypothesis that fluid overload negatively impacts the mucosal barrier, and the questionable impact of ECMO therapy on gut function should be the subject of further studies. 

In a clinical setting, intestinal dysfunction is of subordinate relevance next to cardiac, pulmonary, and cerebral failure and their importance in ECMO therapy. In regard to therapeutic measures, medication with ulinastatin is currently a topic of interest in ischemia-induced intestinal barrier dysfunction [40]. Ulinastatin is a serine protease inhibitor that has anti-inflammatory characteristics and has been widely used as a treatment for patients with inflammatory disorders. Zhang and colleagues could prove a protective role by decreasing oxidative stress in the intestinal mucosa of rats after cardiopulmonary resuscitation. These effects were enlarged by mild therapeutic hypothermia. In particular, this pharmacological approach might be of interest in ECMO-associated gut barrier failure and needs further research. However, our findings and studies discussed above might support and encourage ongoing investigation into this specific subject.

In general, patients receiving veno-arterial ECMO in a clinical scenario suffer some form of cardiogenic/hemodynamic severe compromise associated with capillary leak syndrome. This loss of intravascular volume to the interstitial space may significantly impact the findings of this study. Indeed, this situation may prompt further volume overload, interstitial edema, and, at least, more intestinal mucosa damage (and probably more edema in other organs). This assumption is somehow speculative and must be confirmed in further studies.

### 4.4. Study Limitations

As the study was not powered and designed with regard to the presented topic, results might be limited in this way. Moreover, the methodological classification of volume therapy in MVT and EVT was based on mathematical quantity and was not triggered by pathology-related thresholds. Furthermore, the division of the two cohorts is somehow arbitrary. In a clinical scenario, it may be difficult to objectively identify the point at which patients start to receive a liquid overload. By investigating healthy pigs in a purely experimental and artificial setting, the translation of our findings to clinical scenarios is somehow limited. Furthermore, 10 h of extracorporeal support does not correspond to clinical reality, in which systems are used for a significantly longer time. Therefore, our data cannot answer whether the observation time of 10 h is too short for changes in heart, lung, liver, or kidney tissue or whether fluid overload actually has no effect on these tissues. However, our data might encourage further investigation with a prospectively designed study protocol to address the impact of extensive volume therapy on ECMO circulation more explicitly.

## 5. Conclusions

In our post-hoc investigation of volume therapy’s associated impact on renal function and tissue edema in ECMO circulation, the presented data suggest deterioration of renal function and potential deterioration of intestinal mucosa function by an increase in tissue edema.

## Figures and Tables

**Table 1:** Baseline data prior to ECMO circulation.

Parameter	Moderate Volume Therapy (n = 4)	Extensive Volume Therapy (n = 8)	*p*-Value
Weight (kg)	60 ± 3	59 ± 4	0.670
Hemodynamics			
Cardiac output (L/min)	3.1 ± 0.2	3.0 ± 0.5	0.666
MAP (mmHg)	71 ± 9	66 ± 11	0.417
CVP (mmHg)	12 ± 1	13 ± 4	0.683
RVP_sys_ (mmHg)	32 ± 4	29 ± 9	0.563
PAP_mean_ (mmHg)	25 ± 1	23 ± 3	0.041
LVEDP (mmHg)	7 ± 4	14 ± 12	0.221
Carotidal flow (mL/min)	161 ± 22	153 ± 60	0.738
Respiratory data			
Minute volume (L/min)	6.5 ± 0.4	6.8 ± 1.1	0.398
Horowitz-Index (mmHg)	489 ± 67	475 ± 93	0.933
p_art_O_2_ (mmHg)	116 ± 21	147 ± 38	0.100

(MAP, mean arterial pressure; CVP, central venous pressure; RVP, right ventricular pressure; PAP, pulmonary artery pressure; LVEDP, left ventricular end-diastolic pressure).

**Table 2:** Assessed data after 10 h of ECMO circulation.

Parameter	Moderate Volume Therapy (n = 4)	Extensive Volume Therapy (n = 8)	*p*-Value
Cumulative volume (mL)	3275 ± 263	5344 ± 834	<0.01
Cumulative urine output (mL)	776 ± 495	1003 ± 504	0.484
Hemodynamics			
Cardiac output (L/min)	3.6 ± 0.6	3.3 ± 0.3	0.394
Cardiac Index (L/min/m^2^)	3.3 ± 0.6	3.4 ± 0.3	0.405
MAP (mmHg)	64 ± 7	60 ± 14	0.606
CVP (mmHg)	10 ± 1	11 ± 3	0.727
RVP_sys_ (mmHg)	14 ± 6	23 ± 23	0.808
PAP_mean_ (mmHg)	17 ± 4	18 ± 8	1.000
LVEDP (mmHg)	8 ± 3	14 ± 11	0.181
Flow aorta ascendens (L/min)	0.2 ± 0.1	0.5 ± 0.4	0.128
Coronary flow (mL/min)	16 ± 5	36 ± 25	0.109
Carotidal flow (mL/min)	161 ± 66	179 ± 53	0.655
Respiratory data			
Minute ventilation (L/min)	3.5 ± 0.3	3.2 ± 0.8	0.374
Horowitz-Index (mmHg)	489 ± 67	475 ± 93	0.235
Blood gas			
p_art_O_2_ (mmHg)	119 ± 22	158 ± 53	0.105
Hemoglobin (g/dL)	7.9 ± 0.4	6.7 ± 1.2	0.035
Hematocrit (%)	25 ± 1	21 ± 4	0.036
Na^2+^ (mmol/L)	138 ± 1.7	136 ± 2.5	0.283
K^+^ (mmol/L)	5.9 ± 1.2	6.3 ± 1.2	0.653
Lactate (mmol/L)	1.6 ± 0.6	3.9 ± 2.7	0.046

(MAP, mean arterial pressure; CVP, central venous pressure; RVP, right ventricular pressure; PAP, pulmonary artery pressure; LVEDP, left ventricular end-diastolic pressure).

## Data Availability

Data supporting the reported results can be provided on request by the corresponding author.
